# Classical Swine Fever Virus Infection and Its NS4A Protein Expression Induce IL-8 Production through MAVS Signaling Pathway in Swine Umbilical Vein Endothelial Cells

**DOI:** 10.3389/fmicb.2017.02687

**Published:** 2018-01-12

**Authors:** Wang Dong, Huifang Lv, Kangkang Guo, Tao Wang, Yueling Ouyang, Mingxing Jin, Yanming Zhang

**Affiliations:** College of Veterinary Medicine, Northwest A&F University, Yangling, China

**Keywords:** classical swine fever virus, NS4A, MAVS, ROS, IL-8

## Abstract

Classical swine fever virus (CSFV) infection causes a severe disease of pigs, which is characterized by hemorrhage, disseminated intravascular coagulation, and leucopenia. IL-8, a main chemokine and activator of neutrophils, regulates the permeability of endothelium, which may be related to the hemorrhage upon CSFV infection. Until now, the molecular mechanisms of IL-8 regulation during CSFV infection are poorly defined. Here, we showed that CSFV infection induced IL-8 production and the upregulation of IL-8 required virus replication in swine umbilical vein endothelial cells (SUVECs). Additionally, MAVS expression was increased and was required for IL-8 production upon CSFV infection. Moreover, ROS was involved in CSFV-induced IL-8 production. Subsequent studies demonstrated that ROS was involved in MAVS-induced IL-8 production and CSFV induced ROS production through MAVS pathway. These results indicate that CSFV induces IL-8 production through MAVS pathway and production of ROS. The role of NS4A in the pathogenesis of CSFV is not well-understood. In this study, we further demonstrated that CSFV NS4A induced IL-8 production through enhancing MAVS pathway and promoted CSFV replication. In addition, we discovered that CSFV NS4A was localized in the cell nucleus and cytoplasm, including endoplasmic reticulum (ER) and mitochondria. Taken together, these results provide insights into the mechanisms of IL-8 regulation and NS4A functions during CSFV infection.

## Introduction

Classical swine fever (CSF) is a highly contagious and fatal viral disease against pigs worldwide, which has led to huge economic losses in the swine industry (Dreier et al., 2007; Luo et al., 2014). Classical swine fever virus (CSFV), the causative agent of CSF, is an enveloped RNA virus with a single-stranded positive-sense RNA genome of ~12.3 kb with one large open reading frame (ORF) flanked by 5′ and 3′ untranslated regions (UTRs). CSFV is a member of the *Pestivirus* genus within the *Flaviviridae* family. Hepatitis C virus (HCV) also belongs to this family. The CSFV genome-encoded polyprotein is further processed into 12 mature proteins, which are four structural proteins (C, E^rns^, E1, and E2) and eight non-structural proteins (N^pro^, p7, NS2, NS3, NS4A, NS4B, NS5A, and NS5B) (Thiel et al., 1991; Lamp et al., 2011). CSFV NS4A is an 8 kDa protein, consisting of 64 amino acids, and serves as an essential cofactor for the NS3 protease (Tautz et al., 1997, 2000). Cleaved NS4A is essential for the infectious CSFV particles formation together with NS2-3. In addition, CSFV NS4A interacts with NS3 protein and the downstream nonstructural proteins of the replication complex at the endoplasmic reticulum (ER) membrane where negative-sense RNA templates and progeny viral genomes are produced (Moulin et al., 2007). However, CSFV NS4A is a poorly characterized protein and its roles in the pathogenesis of CSFV are not well-understood.

Interleukin (IL)-8, also known as C-X-C motif ligand (CXCL) 8, is a chemokine that acts as a vital mediator of the innate immunity and has immunomodulatory effects on T-cell function and inflammatory response (Mukaida, 2003). IL-8 is secreted by numerous cells, including monocytes, macrophages, fibroblasts, lymphocytes, neutrophils, endothelial cells, and various normal and malignant epithelial cells (Singh et al., 2013). It is a major mediator of the inflammatory response to numerous bacteria and viruses (Hisatsune et al., 2008; Rajaiya et al., 2008; Zheng et al., 2008; Yu et al., 2011). In addition, IL-8 also plays a crucial role in host defense mechanism by regulating neutrophil activity, but continuous presence of inflammation may cause tissue damage (Dong and Zheng, 2015). Endothelial cells are the major target cells for CSFV and play a key role in maintaining the hemostatic balance (He et al., 2014). Upon to pathogens, endothelial cells are rapidly activated to express pro-inflammatory cytokines to eliminate pathogens. However, pro-inflammatory cytokines may also cause hemostatic balance of the blood vessels. It has been shown that IL-8 regulates the permeability of endothelium by down-regulating tight junction of endothelial cells, which results in vascular disease (Yu et al., 2013). The loss of the endothelial permeability barrier causes hemorrhage (Alam et al., 2012). CSFV infection causes hemorrhagic symptoms of pigs. Therefore, the elevation of IL-8 protein may be related to the hemorrhage upon CSFV infection. Several observations indicate that CSFV infection increases the serum level of IL-8 in 6-month-old pigs (von Rosen et al., 2013) and induces IL-8 expression in swine macrophages (Borca et al., 2008). However, how CSFV induces IL-8 expression and the role of CSFV NS4A protein on IL-8 expression are not yet clear.

Mitochondrial antiviral signaling protein (MAVS) is a crucial common adaptor for retinoic acid-inducible gene I (RIG-I)-like receptors (RLRs) (Sun et al., 2006) and predominantly localizes to the mitochondrial membrane (Wang et al., 2008). During virus infection, RLRs recognize viral RNA and activate MAVS. The activation of MAVS recruits and activates the downstream signaling cascade to induce antiviral and pro-inflammatory factors (Liu et al., 2013, 2015). It has been reported that reactive oxygen species (ROS) production is involved in the induction of IL-8 (Hwang et al., 2004; Ito et al., 2004). However, whether MAVS and ROS are involved in IL-8 induction during CSFV infection is still unknown.

In this study, we showed that CSFV infection induced IL-8 mRNA expression and secretion through MAVS pathway and production of ROS in swine umbilical vein endothelial cells (SUVECs). We also demonstrated that CSFV NS4A induced IL-8 production through enhancing MAVS pathway and NS4A was localized in the cell nucleus and cytoplasm and promoted CSFV replication.

## Materials and methods

### Cells and virus

The established SUVECs were conserved in our laboratory (Hong et al., 2007). The SUVECs were cultured in M199 medium (Gibco, UK) supplemented with 10% fetal bovine serum (FBS) (Biowest, France), 50 μg/mL heparin (Sigma-Aldrich, USA), 100 U/mL penicillin, and 100 mg/mL streptomycin in 5% CO_2_ at 37°C. Human embryonic kidney (HEK293T) cells and swine testicular (ST) cells (ATCC, CRL-1746) were cultured in Dulbecco's minimal essential medium (DMEM) (Gibco, UK) with 10% FBS (Biowest, France) in 5% CO_2_ at 37°C. The CSFV Shimen strain was obtained from the Control Institute of Veterinary Bio-products and Pharmaceuticals (China) and was propagated in ST cells. When the confluency of adherent SUVECs was 70–80%, CSFV was added to the cell culture supernatants at an MOI of 1 for 1 h. Then, the inoculum was removed and the cells were washed three times with PBS to remove unabsorbed virus particles. The cells were added M199 medium containing 2% FBS to culture the infected cells. The cells and supernatants were collected, respectively, at indicated times. All experiments related to CSFV were done in the P3 biosafety laboratory and carried out according to Laboratory Biosafety Manual in our laboratory strictly.

### Plasmid construction and transfection

Total cellular RNA was isolated using TRIzol (Invitrogen, USA) from SUVECs and cDNA was synthesized using First Strand cDNA Synthesis Kit (Thermo, USA). The MAVS gene (GenBank: AB287431.1) was amplified from the cDNA by polymerase chain reaction (PCR) and cloned into the vector pcDNA3.1(–) to generate 3.1-MAVS. Similarly to MAVS, CSFV NS4A gene, and E2 gene were amplified by PCR from the cDNA synthesized from CSFV-infected SUVECs and cloned into the lentivector pCDH-CMV-MCS-EF1 with a Flag-tag to generate CMV-NS4A and CMV-E2, respectively. CSFV NS4A gene was cloned into the vector pEGFP-C1 to generate EGFP-NS4A. Two pairs of shRNA targeting MAVS and a negative control shN (scrambled shRNA) were predicted (http://rnaidesigner.thermofisher.com/) and synthesized. After annealing, the fragments were cloned into pCDH-U6-GreenPuro (SBI, USA) to create MAVS-sh1, MAVS-sh2, and shN lentivectors, respectively. All primers are listed in Table 1. These backbone plasmids of pcDNA3.1(–), pCDH-CMV-MCS-EF1, pEGFP-C1, and pCDH-U6-GreenPuro were conserved in our laboratory. All plasmids were verified by restriction digestion and sequencing. MAVS-sh1, MAVS-sh2, and shN lentivectors were sequenced by specific primer (sequence: TTCTTGGGTAGTTTGCAGTT) and other recombinant plasmids were sequenced by universal primers in the Beijing Genomics Institute (BGI). All plasmids and poly(I:C) (1 μg/mL) (Thermo, USA) were transfected into target cells using the TurboFect Transfection Reagent (Thermo, USA) according to the manufacturer's protocol.

**Table 1:** Primers used in this study.

**Primers**	**Sequence (5′ → 3′)**	**Purpose**
3.1-MAVS-F	GCTCTAGAATGACGTTTGCCGAGGACAAG	Amplification of MAVS
3.1-MAVS-R	ATAAGAATGCGGCCGCTCACTGGGGCAGGCGCCGC	
CMV-NS4A-F	GGAATTCATGTCAACAGCTGAGAATGCCTTG	Amplification of NS4A
CMV-NS4A-R	CGGGATCCTCA**CTTATCGTCGTCATCCTTGTAATC**TAGCTCCTTCAATTCTGTCTCC	
CMV-E2-F	GGAATTCATGCGGCTAGCCTGCAAGGAAGAT	Amplification of E2
CMV-E2-R	CGGGATCCTCA**TTATCGTCGTCATCCTTGTAAT**ACCAGCGGCGAGTTGTTCTGT	
EGFP-NS4A-F	GGAATTCTTCAACAGCTGAGAATGCCTT	Amplification of NS4A
EGFP-NS4A-R	CGGGATCCTCATAGCTCCTTCAATTCTGTCTCC	
IL-8-F	AACTGGCTGTTGCCTTCTTGG	Real-time PCR for detection of IL-8
IL-8-R	GGTGTGGAATGCGTATTTATGC	
CSFV-F	GATCCTCATACTGCCCACTTAC	Real-time PCR for detection of CSFV
CSFV-R	GTATACCCCTTCACCAGCTTG	
β-actin-F	CAAGGACCTCTACGCCAACAC	Real-time PCR for detection of β-actin
β-actin-R	TGGAGGCGCGATGATCTT	
MAVS-sh1-F	GATCCGGATGGATAGCCAGCCTTTCTTCAAGAGAGAAAGGCTGGCTATCCATCCTTTTTG	Knockdown of MAVS
MAVS-sh1-R	AATTCAAAAAGGATGGATAGCCAGCCTTTCTCTCTTGAAGAAAGGCTGGCTATCCATCCG	
MAVS–sh2-F	GATCCGGTGGCTACAGAGAGGATGAGTTCAAGAGACTCATCCTCTCTGTAGCCATTTTTG	Knockdown of MAVS
MAVS–sh2-R	AATTCAAAAATGGCTACAGAGAGGATGAGTCTCTTGAACTCATCCTCTCTGTAGCCACCG	
shN-F	GATCCGCTTAAACGCATAGTAGGACTTCAAGAGAGTCCTACTATGCGTTTAAGCTTTTTG	Knockdown of scrambled
shN-R	AATTCAAAAAGCTTAAACGCATAGTAGGACTCTCTTGAAGTCCTACTATGCGTTTAAGCG	

*Underlines show restriction enzyme sites or loop ring, bolds show flag tag*.

### Lentivirus production

HEK293T cells are commonly used for production of lentivirus, since having the high transfection efficiency and producing high titers of lentivirus. The lentiviruses with MAVS knockdown were produced as previously described method (Lv et al., 2017a). Briefly, HEK293T cells were co-transfected with the plasmids MAVS shRNAs (MAVS-sh1/2) and three other plasmids (pGag/Pol, pRev, pVSVG, conserved in our lab) using TurboFect (Thermo, USA). After 16 h, the culture medium was removed and the fresh advanced DMEM was added with 20 mL/L FBS, 0.01 mM cholesterol (Sigma, USA), 0.01 mM L-α-Phosphatidylcholine (Sigma, USA), 1:1,000 diluted Chemically Defined Lipid (Invitrogen, USA), and 4.0 mM L-Glutamine (Invitrogen, USA). Then, the cells were incubated for 48 h and the culture supernatants containing lentivirus were collected. Lentiviral titers were detected by tissue culture infectious dose (TCID_50_/mL) in HEK293T cells. The lentiviruses were applied to infect SUVECs at 10 transduction units per cell, with polybrene (Sigma, USA) at a final concentration of 6 μg/mL to promote virus attachment. After 8 h, the SUVECs were incubated with fresh medium for another 40 h. The negative control vector (shN) was treated equally.

### Western blot and enzyme-linked immunosorbent assay (ELISA)

The SUVECs were harvested and incubated in radioimmunoprecipitation (RIPA) cell lysis buffer with protease inhibitor phenylmethanesulfonyl fluoride (Beyotime, China) to a final concentration of 1 mM for 30 min on ice. The protein concentration was determined with BCA Protein Assay Kit (Beyotime, China). Protein samples were separated by 12% SDS-PAGE, and then transferred onto polyvinylidene difluoride (PVDF) membranes (Millipore, USA). After blocking with 5% skim milk at room temperature for 2 h, the membranes were incubated with an anti-β-actin mouse monoclonal antibody (Tianjin Sungene Biotech, China) or an anti-MAVS mouse polyclonal antibody at 4°C overnight. Anti-MAVS mouse polyclonal antibody (antigen peptide: residues 150–240 of pig MAVS) was conserved in our laboratory. After five washes with TBST, the membranes were incubated with horseradish peroxidase (HRP)-conjugated goat anti-mouse IgG (1:5,000) secondary antibody (Jackon, USA) for 2 h at room temperature. After another five washes, the signal was detected using an enhanced chemiluminescence (ECL) Western blot analysis system. Secreted IL-8, obtained from cell culture supernatants, was determined using swine ELISA kits (Invitrogen, USA) according to the manufacturer's protocol.

### Real-time PCR

The relative mRNA level of CSFV and IL-8 were tested by real-time PCR with the specific primers in Table 1. Total cellular RNA was extracted with TRIzol (Invitrogen, USA). The cDNA was synthesized using the PrimeScript RT reagent kit (Vazyme, China). The relative mRNA expression was normalized by the housekeeping gene β-actin and was detected with an iCycler iQ5 RealTime Detection System (Bio-Rad, Hercules, CA, USA) using UltraSYBR Mixture (CWBIO, China) according to the manufacturer's protocol. The data were analyzed by the comparative threshold (Ct) method.

### Indirect immunofluorescence assay (IFA)

ST cells are susceptible for CSFV, and CSFV has obviously higher proliferation in ST cells than in other swine cell lines (Li et al., 2014). Thus, ST cells are commonly used to detect the extracellular titers of CSFV (Liang et al., 2017; Lv et al., 2017b). CSFV titers in culture supernatants were determined by IFA as the following methods (Lv et al., 2017a). ST cells in 96-well plate were infected with the progeny viruses by gradient dilution. At 48 h post infection (hpi), the ST cells were first fixed for 20 min at 4°C using stationary liquid. After three washes with PBS, the cells were penetrated for 20 min at 4°C 1% tritonx-100. Followed by another three washes, the cells were blocked for 2 h at 37°C with 10% skim milk. Then, the cells were incubated with positive CSFV serum conserved in our lab at 4°C overnight. Followed by five washes with PBST, the cells were incubated with rabbit anti-pig IgG-FITC antibody (Sigma, USA) for 2 h at 37°C. After another five washes with PBST, Nuclei were stained with 4′,6-diamidino-2-phenylindole (DAPI). Fluorescence was observed under a fluorescence microscope (Nikon, Japan). Mock-infected cells were used as negative controls.

### Detection of intracellular ROS levels

The levels of intracellular ROS were determined by an oxidation-sensitive fluorescent probe DCFH-DA or dihydroethidium (DHE) (Beyotime). After CSFV infection and 3.1-MAVS transfection and N-acetyl-L-cysteine (NAC) (Beyotime) treatment for the specified time points, SUVECs were harvested and treated with 10 μM DCFH-DA or 2.5 μM DHE for 20 min at 37°C. H_2_O_2_ was served as a control. Cells were washed with PBS, and ROS fluorescence was detected using VICTOR X3 Multimode Plate Reader (Perkin Elmer, America). SUVECs were infected with lentivirus-MAVS-sh1 or lentivirus-shN (generated a GFP reporter) for 24 h, then the cells were infected with CSFV for an additional 36 h. The levels of intracellular ROS were detected using the fluorescent probe DHE (suitable for the cells expressing GFP).

### Confocal microscopy

SUVECs were seeded on the glass coverslips of the 35 mm diameter cell culture dishes and cultured to 20–30% confluency. Then, SUVECs were transfected with pEGFP-C1 or pEGFP-NS4A. The cells were further cultured for 36 h. Then, the cells were washed three times and were incubated with 1 μM ER-tracker Red (Beyotime, China) for 30 min at 37°C or incubated with 200 nM Mito-Tracker Red CMXRos (Yeasen, China) for 45 min at 37°C and washed with PBS for three times. Then, the cells were fixed with 4% paraformaldehyde for 20 min at room temperature and incubated with Hoechst 33342 for 10 min at room temperature. After three washes with PBS, cells were observed by laser scanning confocal microscopy (LSM510 META; Zeiss, Germany). ER-Tracker Red is a red fluorescent probe of ER and used for marking the location of intracellular ER. Mito-Tracker Red CMXRos is a red oxidized fluorescent dye of mitochondria and used for marking the location of intracellular mitochondria. Hoechst 33342 is a blue fluorescent dye of nuclear DNA and used for marking the location of cell nucleus.

### Cell viability assay

To evaluate the effects of IL-8 (novoprotein, CC97) on the growth of SUVECs, cell viability was measured by the cell counting kit-8 (CCK-8; Beyotime, C0038) according to the manufacturer's instructions.

### Statistical analysis

All experiments were performed at least three times, and the results represent the mean ± standard deviation (SD) of three replicates. The data are analyzed by one-way ANOVA and Bonferroni post-hoc test using the SPSS software (version 18.0). A *P* < 0.05 was considered significant.

## Results

### CSFV infection induces IL-8 production

**Figure 1:**
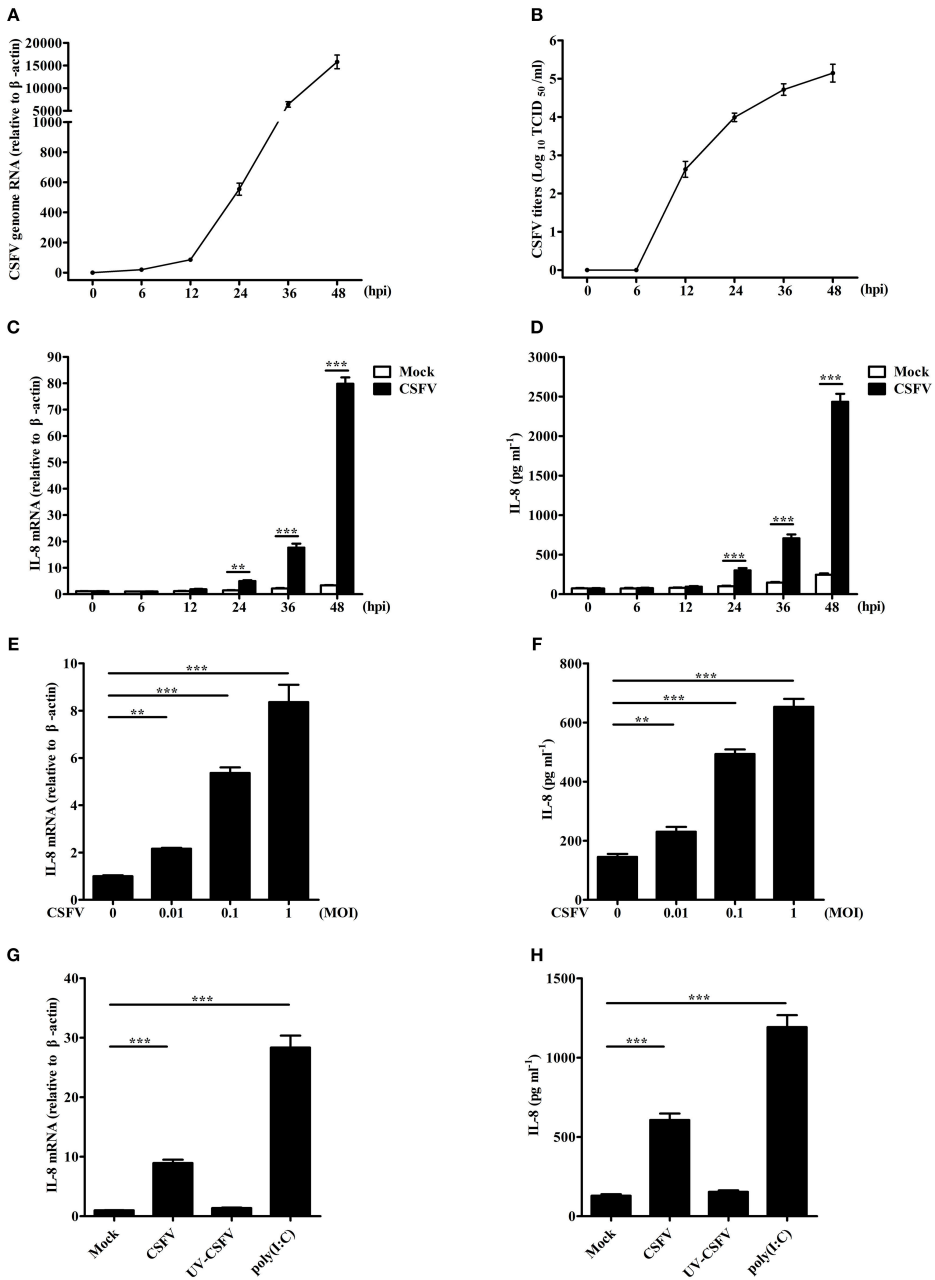
CSFV infection induces IL-8 production. **(A,B)** The growth curve of CSFV. SUVECs were infected with CSFV at an MOI of 1. The supernatants, which contain progeny viruses, and cells were collected at 0, 6, 12, 24, 36, and 48 hpi, respectively. CSFV genome RNA was determined by real-time PCR. The extracellular viral titers were detected and expressed as TCID_50_/mL. The supernatants at 0 hpi was the supernatants of SUVECs infected without CSFV at 0 h. **(C–F)** IL-8 mRNA expression and secretion in CSFV-infected SUVECs. SUVECs were infected with CSFV at an MOI of 1 and IL-8 mRNA expression and secretion were determined at 0, 6, 12, 24, 36, and 48 hpi **(C,D)**, or infected at an MOI of 0, 0.01, 0.1, and 1 and IL-8 mRNA expression and secretion was determined at 36 hpi **(E,F)** by real-time PCR and ELISA. **(G,H)** IL-8 mRNA expression and secretion in UV-inactivated CSFV-infected SUVECs. SUVECs were infected with CSFV or UV-inactivated CSFV at an MOI of 1 for 36 h or were transfected with poly(I:C) (1 μg/mL) for 12 h and analyzed for IL-8 production by real-time PCR and ELISA. Error bars represent the mean ± *SD* (*n* = 3). ^**^P < 0.01; ^***^P < 0.001.

To investigate the effect of CSFV on IL-8 transcription and secretion, SUVECs were infected with CSFV. Firstly, life cycle of CSFV in SUVECs was detected, and the growth curve of CSFV was built (Figures 1A,B). In addition, CSFV infection up-regulated IL-8 mRNA expression at 24, 36, and 48 hpi (Figure 1C). We also found increased secretion of IL-8 at 24, 36, and 48 hpi compared to the mock-infected controls (Figure 1D), implying the increase of IL-8 expression might be correlated with CSFV growth. Furthermore, CSFV infection increased IL-8 production at a multiplicity of infection (MOI) of 0.01, 0.1, or 1 at 36 hpi (Figures 1E,F). These data indicate that CSFV induces IL-8 production in a dose dependent manner. To detect whether the upregulation of IL-8 was dependent on viral replication, SUVECs were infected with UV-inactivated CSFV or transfected with poly(I:C), a synthetic analog of double-stranded RNA. The results showed that the treatment of poly(I:C) significantly increased IL-8 production. However, UV-inactivated CSFV did not induce IL-8 production (Figures 1G,H). Collectively, these findings suggest that CSFV infection induces IL-8 production and the upregulation of IL-8 requires CSFV replication.

### MAVS is involved in CSFV-induced IL-8 production

**Figure 2:**
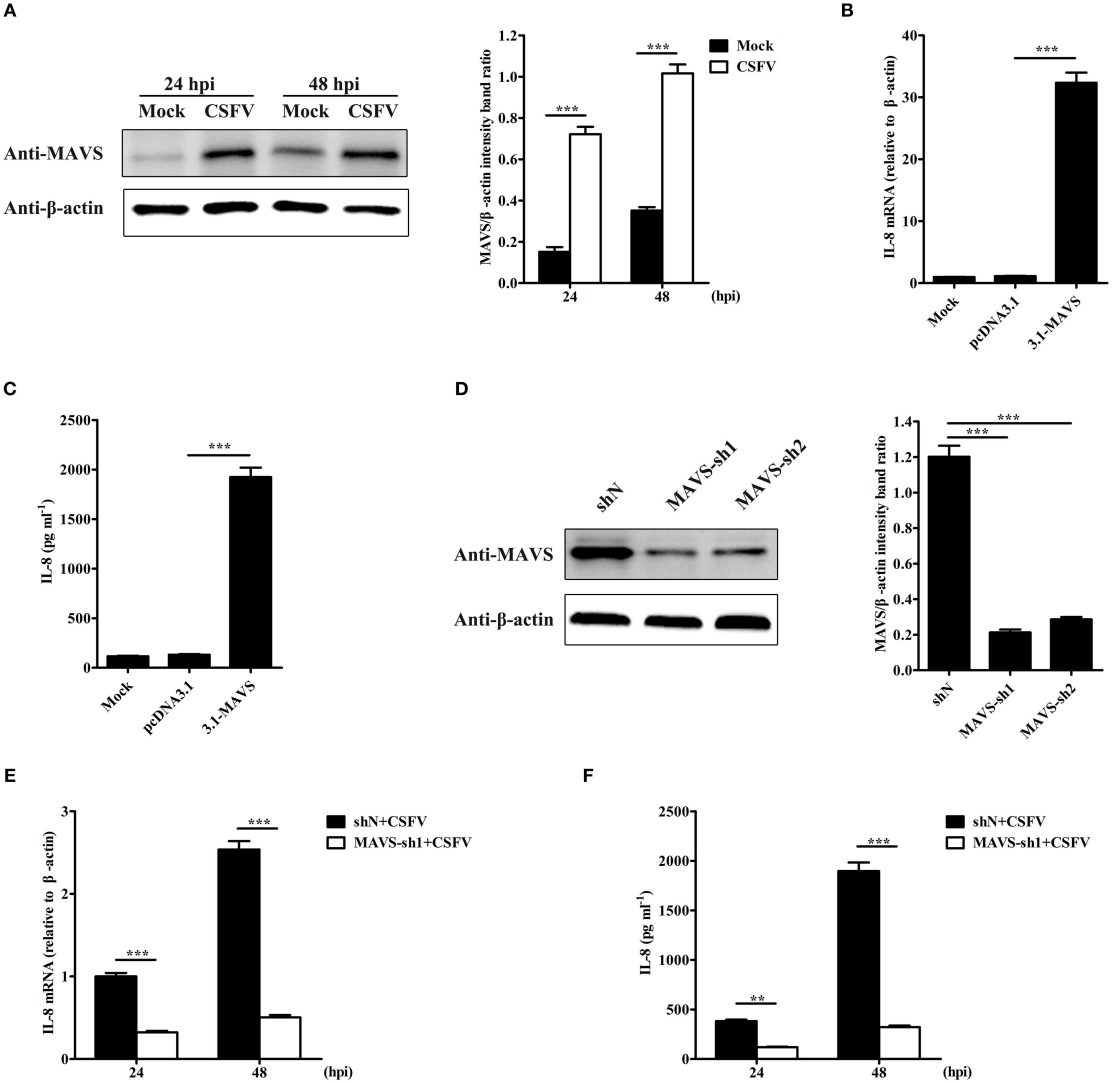
MAVS is involved in CSFV-induced IL-8 production. **(A)** MAVS expression in CSFV-infected SUVECs. SUVECs were infected with CSFV at an MOI of 1 and MAVS expression was determined at 24 and 48 hpi by Western blot. **(B,C)** IL-8 mRNA expression and secretion in MAVS-overexpressing SUVECs. SUVECs were transfected with 3.1-MAVS for 30 h and analyzed for IL-8 production by real-time PCR and ELISA. **(D)** Knockdown of MAVS by shRNAs. SUVECs were infected with shN or MAVS-sh1/2 lentivirus. Cells were collected and the knockdown efficiency of MAVS was assessed by Western blot at 48 hpi. **(E,F)** IL-8 mRNA expression and secretion in MAVS-knockdown SUVECs infected with CSFV. SUVECs were infected with shN or MAVS-sh1 lentivirus. At 24 hpi, cells were infected with CSFV at an MOI of 1 for an additional 24 and 48 h. IL-8 production was analyzed by real-time PCR and ELISA. Error bars represent the mean ± *SD* (*n* = 3). ^**^P < 0.01; ^***^P < 0.001.

To detect the signaling pathways involved in CSFV-induced IL-8 production, the role of MAVS signaling pathway was examined. As shown in Figure 2A, MAVS protein level was significantly increased in CSFV-infected SUVECs at an MOI of 1 at 24 and 48 hpi. In addition, MAVS overexpression markedly up-regulated the mRNA expression and secretion of IL-8 (Figures 2B,C). We hypothesized that MAVS pathway may be involved in CSFV-induced IL-8 production. To verify this, two pairs of specific small hairpin RNA (shRNA) were used to silence endogenous MAVS expression in SUVECs by lentivirus infection. The Western blot results showed that the knockdown efficiency of MAVS-sh1 cells was highest (Figure 2D), and the cells were thus used for subsequent experiment. SUVECs were infected with MAVS-sh1-lentivirus or shN-lentivirus for 24 h. Then, the cells were infected with CSFV at an MOI of 1. Compared with shN-treated cells, IL-8 production was significantly reduced in MAVS-sh1-treated cells at 24 and 48 hpi (Figures 2E,F). Together, the data indicate that CSFV infection induces IL-8 expression through activating the MAVS pathway.

### ROS is involved in CSFV-induced IL-8 production

**Figure 3:**
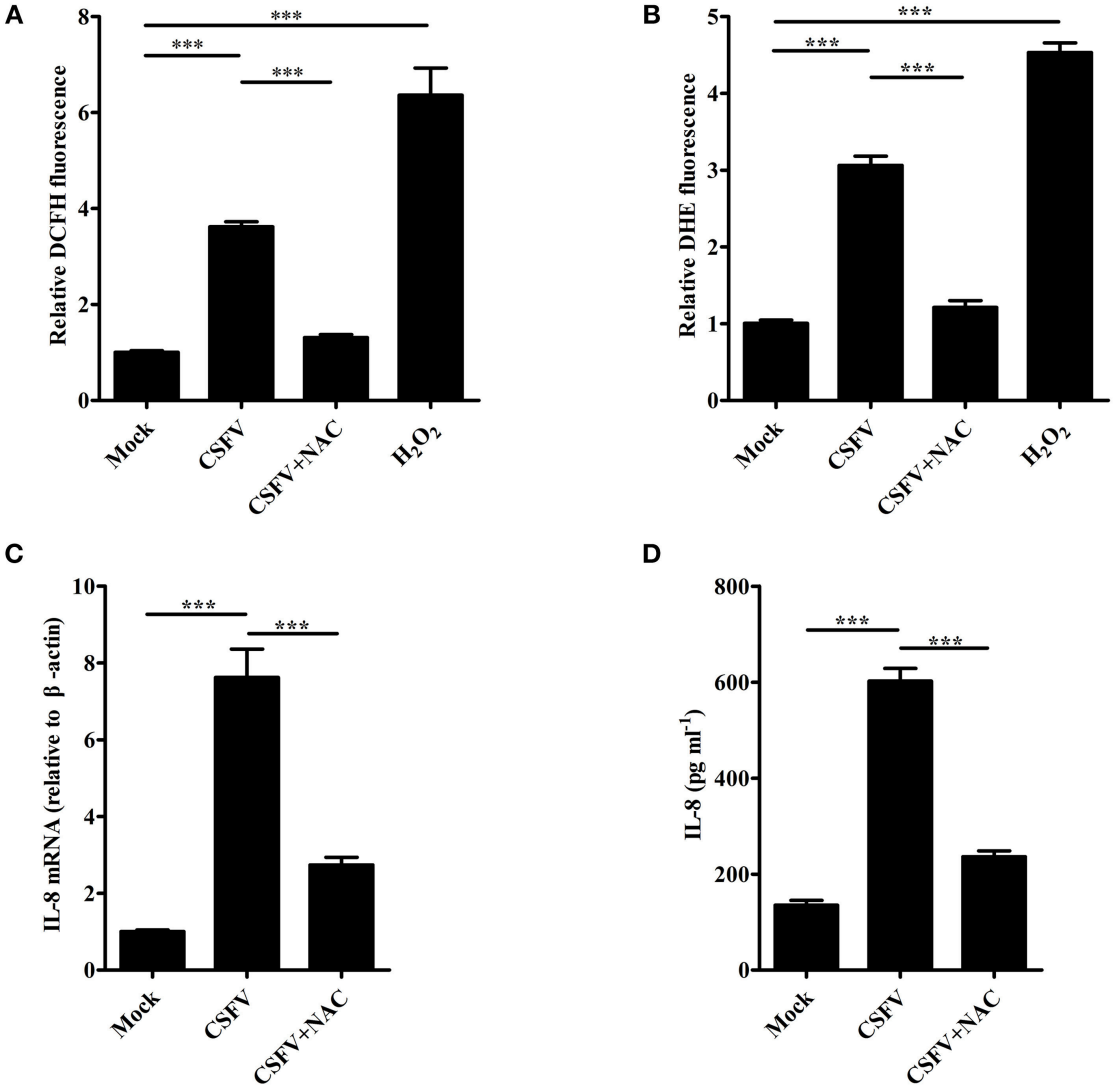
ROS is involved in CSFV-induced IL-8 production. **(A,B)** The ROS levels in CSFV-infected SUVECs. SUVECs were infected with CSFV at an MOI of 1 and treated with or without NAC (3 mM) for 36 h. The intracellular ROS levels were measured by the fluorescent probe DCFH-DA and DHE. H_2_O_2_ (40 μM) was used as a positive control. **(C,D)** IL-8 mRNA expression and secretion in CSFV-infected SUVECs with NAC treatment. SUVECs were infected with CSFV at an MOI of 1 and treated with or without NAC for 36 h and IL-8 production was analyzed by real-time PCR and ELISA. Error bars represent the mean ± *SD* (*n* = 3). ^***^P < 0.001.

It has been reported that ROS production participates in the induction of IL-8 (Ito et al., 2004). To investigate whether ROS is involved in CSFV-induced IL-8 production, SUVECs were infected with CSFV and treated with NAC, an antioxidant. Firstly, intracellular ROS level was analyzed at 36 hpi. As shown in Figure 3A, CSFV infection or H_2_O_2_ treatment increased ROS production and the treatment of NAC significantly reduced CSFV-induced ROS production by using the fluorescent probe DCFH-DA in SUVECs. The same results were observed by using another fluorescent probe DHE (Figure 3B). In addition, the mRNA expression and secretion of IL-8 were measured in CSFV-infected SUVECs treated with NAC. Importantly, NAC treatment significantly reduced CSFV-induced IL-8 mRNA expression and secretion (Figures 3C,D). These data indicate that ROS is involved in CSFV-induced IL-8 production.

### CSFV induces IL-8 production through MAVS pathway and production of ROS

**Figure 4:**
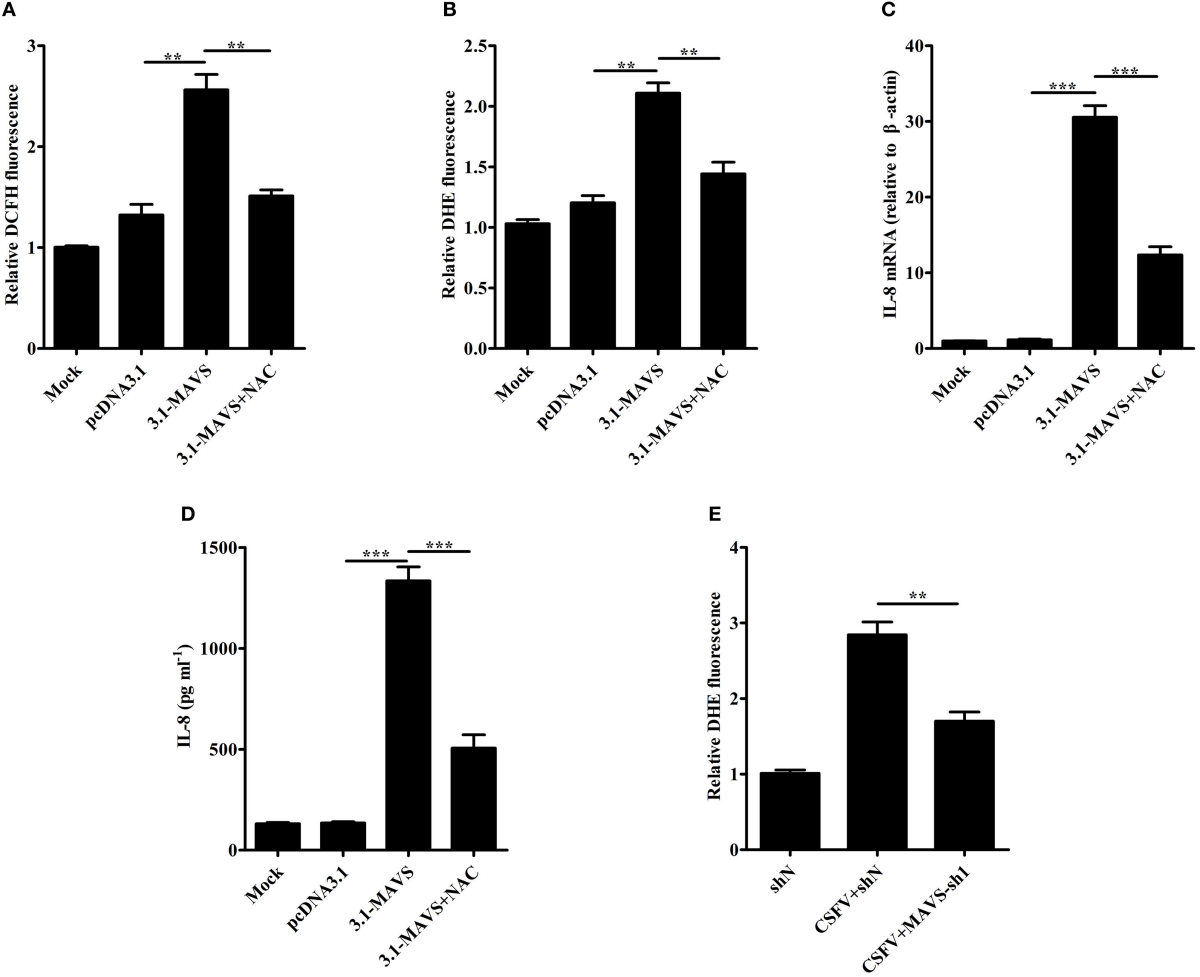
CSFV induces IL-8 production through MAVS pathway and production of ROS. **(A,B)** The ROS levels in MAVS-overexpressing SUVECs. SUVECs were transfected with 3.1-MAVS and treated with or without NAC for 30 h. The intracellular ROS levels were measured by the fluorescent probe DCFH-DA and DHE. **(C,D)** IL-8 mRNA expression and secretion in MAVS-overexpressing SUVECs with NAC treatment. **(E)** The ROS levels in MAVS-knockdown SUVECs infected with CSFV. SUVECs were infected with lentivirus-MAVS-sh1 or lentivirus-shN for 24 h, and the cells were infected with or without CSFV for an additional 36 h. The intracellular ROS levels were measured by the fluorescent probe DHE. Error bars represent the mean ± *SD* (*n* = 3). ^**^P < 0.01; ^***^P < 0.001.

To study whether the increase of ROS production is related to MAVS, SUVECs were first transfected with 3.1-MAVS and treated with NAC, then intracellular ROS level was analyzed at 30 h post transfection (hpt). The results showed that overexpression of MAVS increased ROS production. However, MAVS-mediated ROS level was inhibited by the treatment of NAC (Figures 4A,B). To further investigate the effect of ROS on MAVS-induced IL-8, the IL-8 mRNA expression and secretion were measured in MAVS-transfected SUVECs treated with NAC. The results showed that NAC treatment reduced MAVS-induced IL-8 production at both mRNA and protein levels (Figures 4C,D). These results demonstrated that overexpression of MAVS increased ROS production and ROS was involved in MAVS-induced IL-8 production, indicating that the enhancement of IL-8 expression was associated with MAVS-mediated ROS. To further investigate whether CSFV induced ROS production through MAVS pathway, intracellular ROS level was measured in MAVS-knockdown SUVECs infected with CSFV. As shown in Figure 4E, the knockdown of MAVS reduced CSFV-induced ROS. Taken together, these data suggest that CSFV induces IL-8 production through MAVS pathway and production of ROS.

### Subcellular localization of CSFV NS4A protein

**Figure 5:**
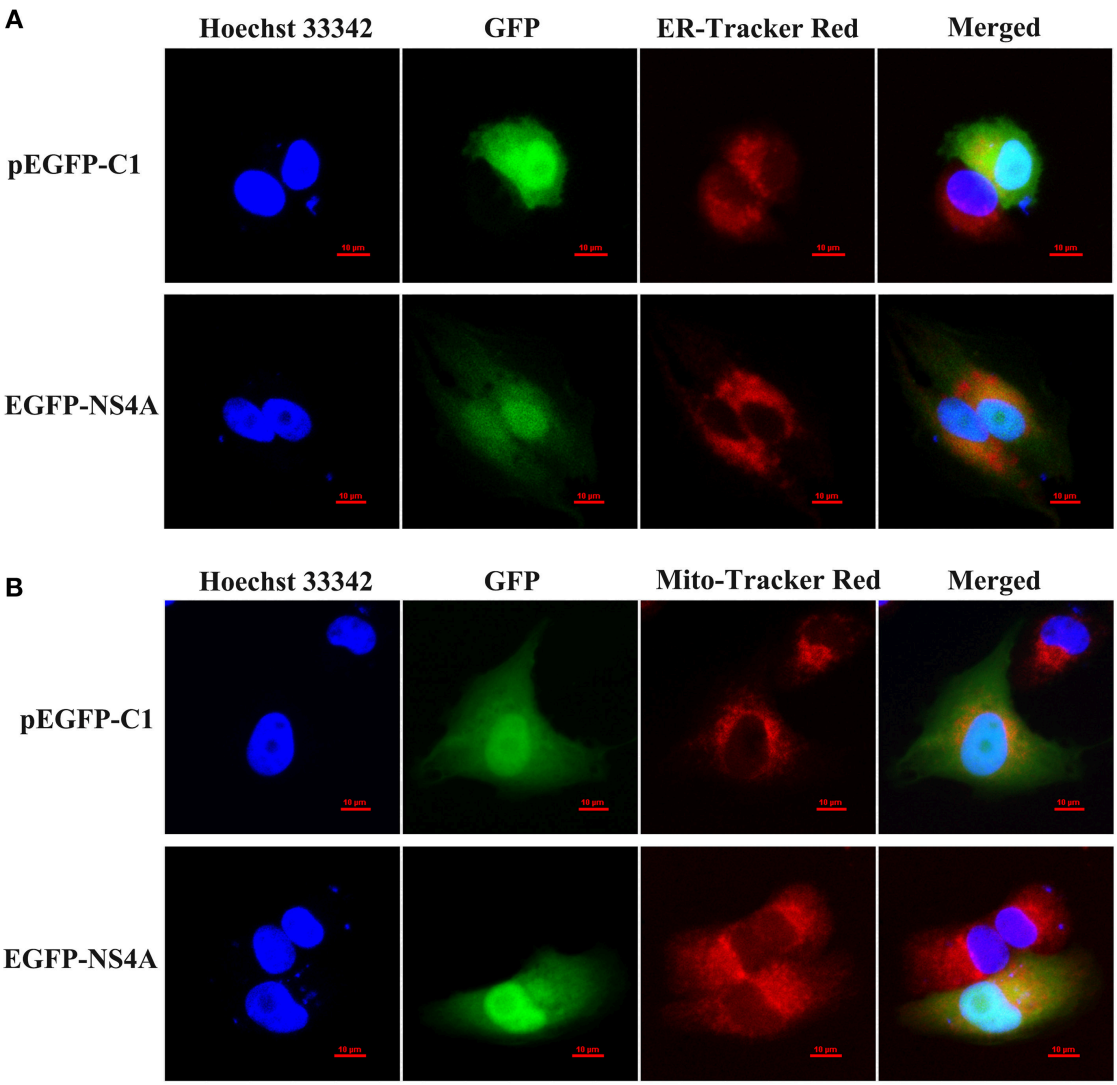
Subcellular localization of EGFP-NS4A fusion protein in SUVECs. The EGFP-NS4A fusion protein subcellular localization was detected by confocal microscopy. All cells were stained with Hoechst 33342 and ER-Tracker Red **(A)** or Mito-Tracker Red **(B)**. Scale bar = 10 μm. The location of intracellular ER is indicated by ER-Tracker Red and shows red fluorescent. The location of intracellular mitochondria is indicated by Mito-Tracker Red and shows red fluorescent. The location of cell nucleus is indicated by Hoechst 33342 and shows blue fluorescent.

CSFV NS4A is a non-structural protein and is poorly studied. To further clarify the function of NS4A in CSFV pathogenesis, the subcellular localization of NS4A was detected by confocal fluorescence microscopy. As shown in Figure 5A, EGFP-NS4A was distributed in the cell nucleus and cytoplasm, implying that CSFV NS4A might regulate various intracellular biochemical events. To further confirm cytoplasmic distribution of NS4A, we examined whether NS4A located in ER or mitochondria. NS4A was observed in both ER and mitochondria (Figures 5A,B). These results suggest that CSFV NS4A may regulate the function of some proteins of ER and mitochondria and contribute to a variety of pathogenesis of CSFV.

### CSFV NS4A induces IL-8 production

**Figure 6:**
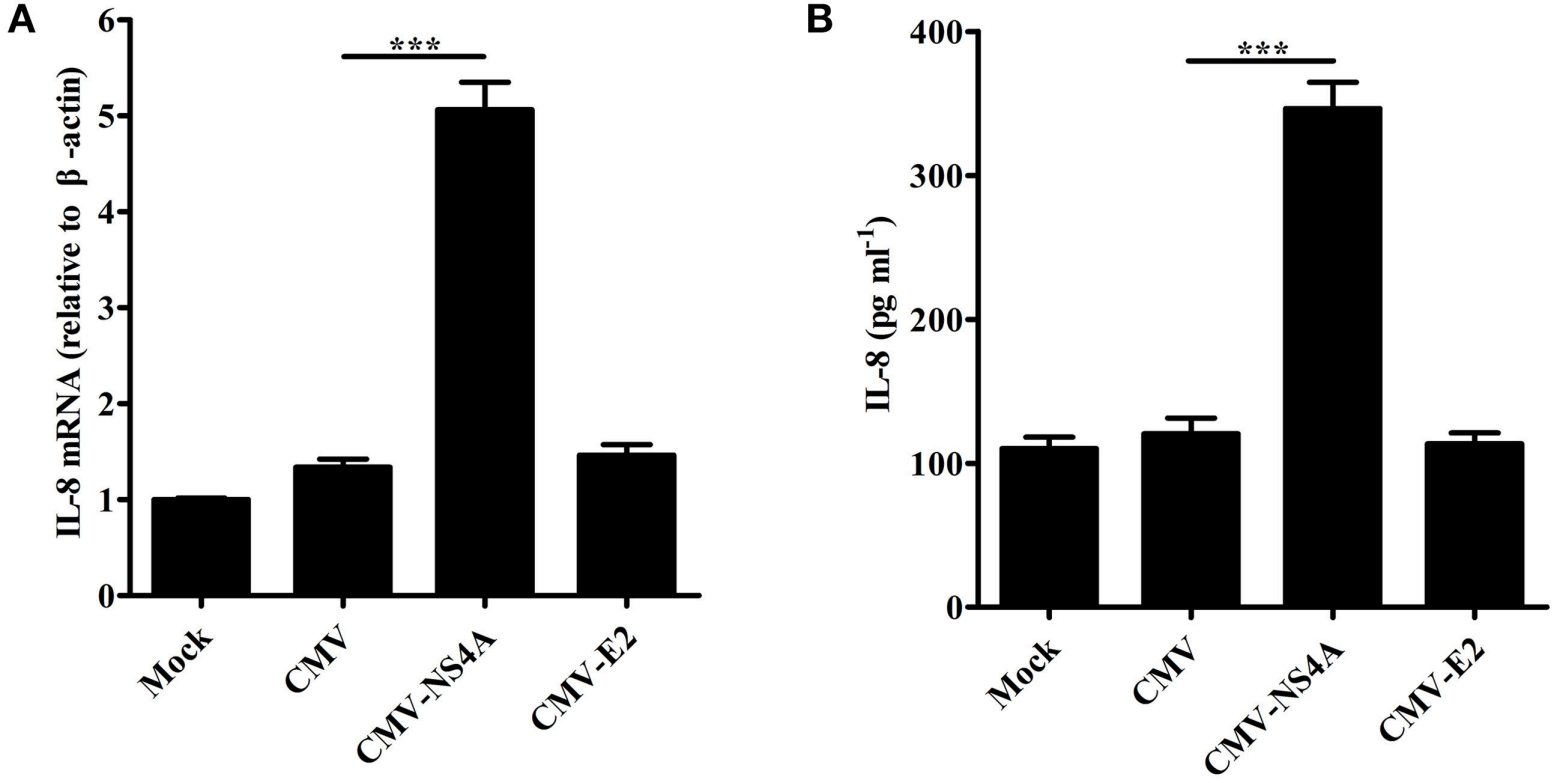
CSFV NS4A induces IL-8 production. **(A,B)** IL-8 mRNA expression and secretion in NS4A-transfected SUVECs. SUVECs were transfected with CMV-NS4A or CMV-E2 for 30 h and analyzed for IL-8 production by real-time PCR and ELISA. Error bars represent the mean ± *SD* (*n* = 3). ^***^P < 0.001.

To explore the effect of CSFV NS4A on IL-8 transcription and secretion, SUVECs were transfected with CMV-NS4A or CMV-E2, and IL-8 expression was analyzed at 30 hpt. As shown in Figures 6A,B, increased mRNA expression and secretion of IL-8 were observed in NS4A-transfected cells. However, CSFV E2 did not alter the expression of IL-8. These results indicate that CSFV NS4A induces IL-8 production.

### NS4A induces IL-8 production through enhancing MAVS pathway

**Figure 7:**
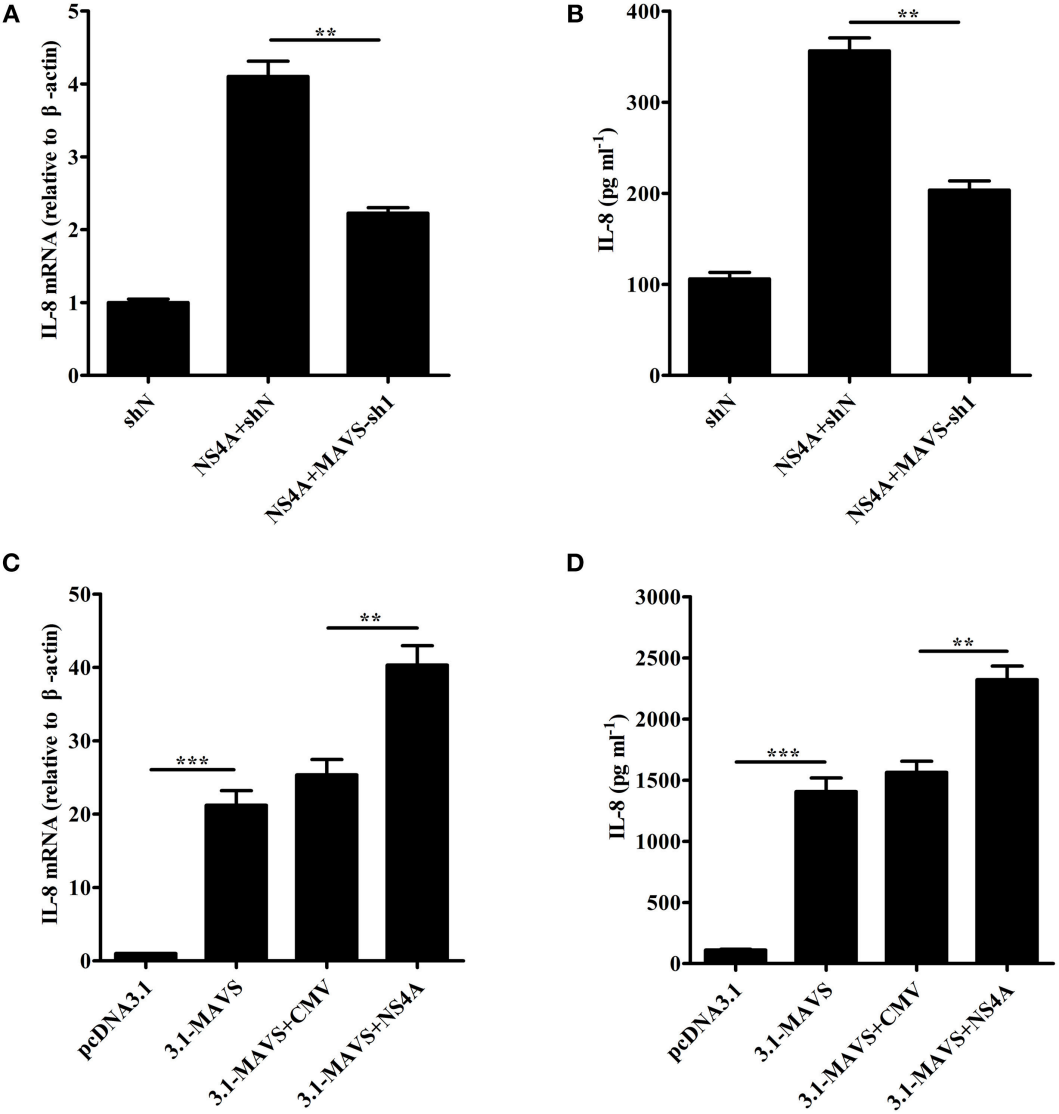
NS4A induces IL-8 production through enhancing MAVS pathway. **(A,B)** The knockdown of MAVS reduced NS4A-induced IL-8 production. SUVECs were infected with shN or MAVS-sh1 lentivirus. At 24 hpi, cells were transfected with CMV-NS4A for an additional 30 h. IL-8 production was analyzed by real-time PCR and ELISA. **(C,D)** NS4A enhanced MAVS-induced IL-8 production. SUVECs were co-transfected with CMV-NS4A and 3.1-MAVS for 30 h and analyzed for IL-8 production by real-time PCR and ELISA. Error bars represent the mean ± *SD* (*n* = 3). ^**^P < 0.01; ^***^P < 0.001.

To study the role of MAVS in NS4A-induced IL-8 production, we examined the levels of IL-8 induced by NS4A in MAVS-knockdown SUVECs. As shown in Figures 7A,B, the knockdown of MAVS reduced NS4A-induced IL-8 mRNA expression and secretion. These results indicate that MAVS is required to NS4A-induced IL-8 production. To further investigate the mechanism of NS4A-induced IL-8 production, we checked the role of NS4A in MAVS-induced IL-8 production. The results showed that NS4A enhanced MAVS-induced IL-8 production (Figures 7C,D). These data indicate that NS4A induces IL-8 production through enhancing MAVS pathway.

### NS4A promotes CSFV propagation

**Figure 8:**
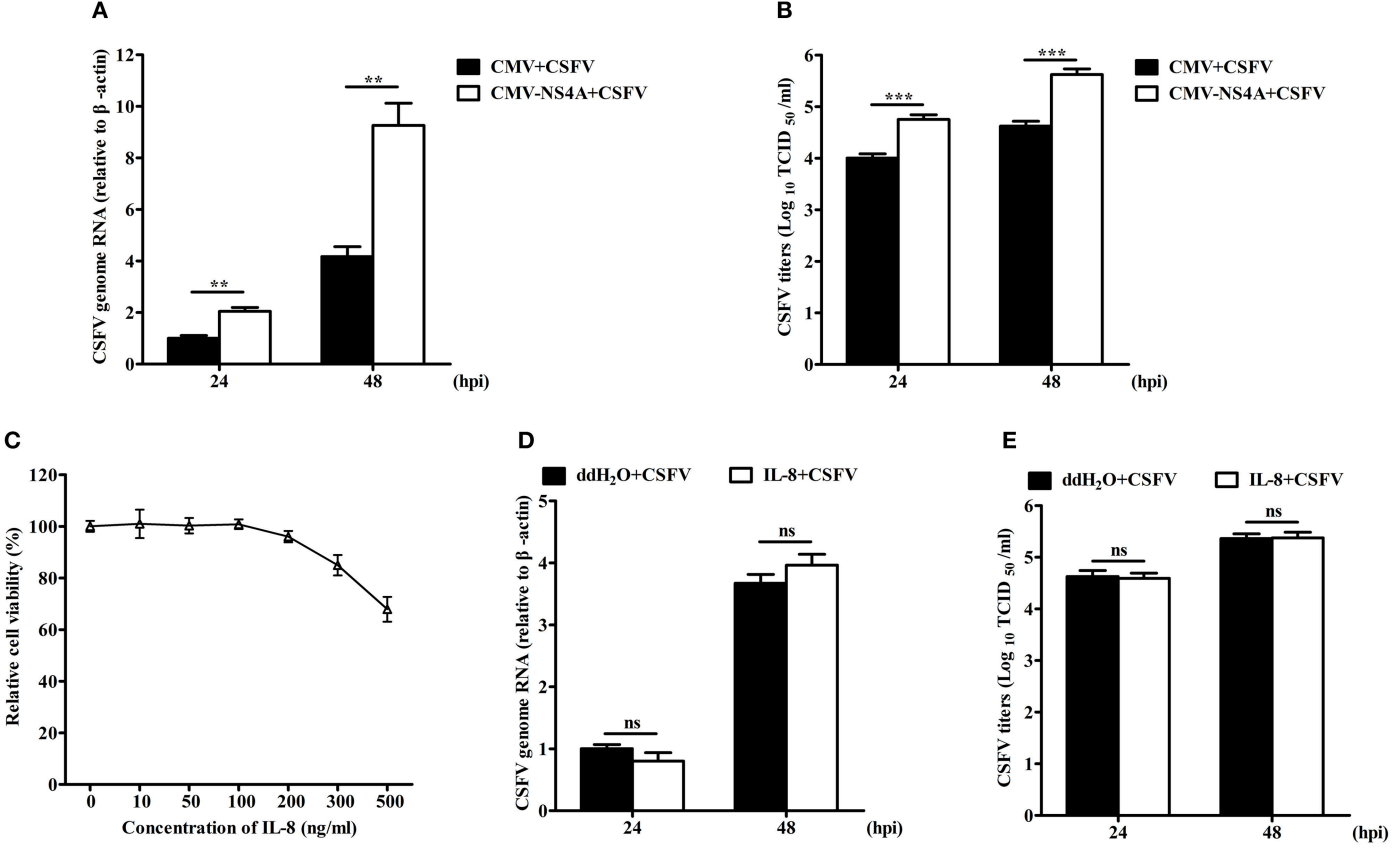
NS4A promotes CSFV propagation. **(A,B)** CSFV propagation in NS4A-transfected SUVECs. SUVECs were transfected with CMV-NS4A. At 12 hpt, cells were infected with CSFV at an MOI of 0.1 for 24 and 48 h. Cells were collected and CSFV genome RNA was determined by real-time PCR. The supernatants were collected and the extracellular viral titers were detected and expressed as TCID_50_/mL at 24 and 48 hpi. **(C)** The cell viability upon the treatment of IL-8. SUVECs were treated with different concentrations of IL-8 for 48 h and the cell viability was measured by the CCK-8 assay. **(D,E)** CSFV propagation in IL-8-treated SUVECs. SUVECs were infected with CSFV at an MOI of 0.1 and maintained in media with IL-8 (100 ng/mL). Then CSFV genome RNA and extracellular viral titers were determined by real-time PCR and IFA. Error bars represent the mean ± *SD* (*n* = 3). ^**^P < 0.01; ^***^P < 0.001; ns (no significant), *P* > 0.05.

To investigate the effect of NS4A on CSFV replication, we detected the virus growth in NS4A-transfected SUVECs. CSFV genome RNA and the extracellular titers of progeny virus were increased in SUVECs overexpressing NS4A, compared to those in SUVECs expressing empty vector at 24 and 48 hpi (Figures 8A,B). To further study whether IL-8 contributed to CSFV replication, we first examined the cytotoxicity of IL-8 on SUVECs. Cells viability showed that the safe concentration of IL-8 to SUVECs was 100 ng/mL (Figure 8C). Then SUVECs were infected with CSFV at an MOI of 0.1 and maintained in media with or without IL-8 (100 ng/mL). As shown in Figures 8D,E, CSFV genome RNA and the extracellular titers of progeny virus were not changed in IL-8-treated cells. These results indicate that IL-8 cannot alter CSFV replication, implying that NS4A may promote CSFV propagation in an IL-8-independent manner.

## Discussion

IL-8 is a major chemokine and activator of neutrophils, which is involved in various non-specific pathological processes of inflammation (Pease and Sabroe, 2002). Previous studies have indicated that IL-8 regulates the permeability of endothelium, which may cause hemorrhagic symptoms (Alam et al., 2012; Yu et al., 2013). Given hemorrhagic symptoms of pigs upon CSFV infection, whether IL-8 production is induced upon CSFV infection and the mechanism of IL-8 expression are important for understanding the pathology of CSFV infection. Here, we demonstrated that CSFV infection induced IL-8 production in a dose dependent manner in SUVECs (Figures 1A–F), similar to that CSFV infection up-regulates IL-8 levels in swine macrophages and *in vivo* (Borca et al., 2008; von Rosen et al., 2013). Moreover, we demonstrated that the upregulation of IL-8 required CSFV replication. In addition, poly(I:C) increased IL-8 expression (Figures 1G,H), suggesting CSFV RNA may play an important role for CSFV-induced IL-8 production.

Although CSFV infection induces IL-8 production, how CSFV induces IL-8 production remains unclear. CSFV can be recognized by RLRs to induce antiviral and pro-inflammatory factors (Dong et al., 2013). MAVS is a crucial common adaptor for RLRs (Sun et al., 2006). Here, we demonstrated that CSFV induces IL-8 production through MAVS signaling pathway for the first time (Figure 2). ROS production has been shown to participate in IL-8 induction (Hwang et al., 2004; Ito et al., 2004). Intracellular ROS is elevated under various physiological and pathological conditions (Ali et al., 1999). In a previous study, CSFV infection stimulated ROS accumulation in 3D4/2 macrophages (Lin et al., 2014). Thus, whether ROS was involved in the induction of IL-8 production by CSFV infection was further verified. Here, we demonstrated that CSFV infection increased ROS production in SUVECs. Importantly, the treatment of antioxidant NAC reduced CSFV-induced IL-8 production (Figure 3), indicating that ROS was involved in CSFV-induced IL-8 production.

Cellular ROS is generated as a byproduct of mitochondrial oxidative metabolism, and xenobiotics, such as cytokines and bacterial invasion also stimulate formation of ROS (Zhang et al., 2016). Here, we found that MAVS overexpression increased ROS production and the treatment of NAC reduced MAVS-induced IL-8 production (Figures 4A–D), indicating that ROS is involved in MAVS-induced IL-8 production. Given the increase of MAVS expression and ROS production upon CSFV infection, we speculated CSFV induced ROS production through MAVS signaling pathway. As we expected, the knockdown of MAVS reduced CSFV-induced ROS production (Figure 4E). Taken together, these results demonstrate that CSFV induces IL-8 production through MAVS pathway and production of ROS. The downstream NF-κB, Mitogen-activated protein kinase (MAPK) ERK, p38, JNK, and PI3K-Akt are involved in the regulation of IL-8 expression (Li et al., 2002; Chen et al., 2015; Wang et al., 2015; Yi et al., 2015; Liu et al., 2017). In addition, the increase of ROS impacts on the activation of NF-κB, MAPK ERK, JNK, p38, and PI3K-Akt signaling pathways (Thannickal and Fanburg, 2000; Kyaw et al., 2001; Qadri et al., 2004; Groeger et al., 2009; Yi et al., 2015; Zhang et al., 2016). However, CSFV fails to activate the NF-κB-signaling pathway (Chen et al., 2012; Cao et al., 2015). Thus, we speculated that CSFV may induce IL-8 production through ROS-mediated ERK, JNK, p38, or PI3K-Akt pathways, and the hypothesis will be further studied in future research. A previous study shows that H_2_O_2_ treatment slightly enhances basal IL-8 production in human airway epithelial cells (Ito et al., 2004). In this study, we demonstrated that the reduction of intracellular ROS down-regulated CSFV-induced IL-8 production. These data indicate that ROS is involved in CSFV-induced IL-8 production, but ROS itself may not up-regulate the production of IL-8 directly. Similarly, H_2_O_2_ treatment markedly potentiates IL-1β-stimulated IL-8 production (Ito et al., 2004).

CSFV NS4A serves as an essential cofactor for the NS3 protease (Tautz et al., 1997, 2000). NS4A and NS2-3 play an important role for infectious particle formation (Moulin et al., 2007). HCV NS4A is located in the ER as well as mitochondria (Dabral et al., 2014). It has been reported that HCV NS4A expression lead to the alteration of intracellular distribution of mitochondria and its damage (Nomura-Takigawa et al., 2006). In this study, we also revealed that CSFV NS4A was distributed in the whole cells, including cell nucleus, ER, and mitochondria (Figure 5). The location of cell nucleus suggests that NS4A may regulate the transcription of some intracellular proteins to promote CSFV replication. The location of ER verifies that NS4A associates with other CSFV proteins to complete the assembly of viral replication complex and suggests that CSFV NS4A may regulate the function of some proteins of ER. The location of mitochondria implies that NS4A may alter mitochondrial metabolism or impact on the function of some mitochondrial proteins. Our further studies showed that CSFV NS4A induced IL-8 production but CSFV E2 did not induce IL-8 production (Figure 6), indicating that NS4A may play an important role in CSFV-induced IL-8 production and NS4A-induced IL-8 production may be associated with pathogenesis of CSFV. MAVS predominantly localizes to the mitochondrial membrane (Wang et al., 2008). Given MAVS-induced IL-8 production, we detected the role of MAVS in NS4A-induced IL-8 production. The result showed that MAVS was required for NS4A-induced IL-8 production. Notably, we observed that NS4A enhanced MAVS-induced IL-8 production (Figure 7), indicating that NS4A induced IL-8 production through enhancing MAVS pathways. Therefore, we speculate that CSFV NS4A may be localized in the mitochondrial membrane and activate MAVS signaling pathway, ultimately inducing IL-8 production. Further studies will be done to verify the content and whether other proteins of CSFV induce IL-8 production as well as whether ROS is involved in NS4A-induced IL-8 production.

NS4A plays an essential role in HCV replication by regulating NS5A phosphorylation, and its expression is also required for NS5A hyperphosphorylation (Tanji et al., 1995). In addition, HCV NS4A plays an important role for the viral life cycle (Hara et al., 2009). Therefore, we detected the role of NS4A in CSFV replication. We found that NS4A overexpression increased CSFV replication in SUVECs (Figure 8). However, the increased fold of extracellular progeny virus was higher than that of CSFV genome RNA in SUVECs overexpressing NS4A, implying that CSFV NS4A might play major roles in translation, assembly or release of CSFV particles. Similarly, CSFV NS4A plays an important role for infectious particle formation (Moulin et al., 2007). However, the hypothesis needs to be further verified in future studies. IL-8 has also been reported to be an important mediator of the innate immunity (Mukaida, 2003). IL-8 may antagonize the antiviral effect of IFN-α (Khabar et al., 1997). Given NS4A-induced IL-8 production, we detected whether the upregulation of IL-8 production contributed to CSFV replication. Interestingly, we found that IL-8 had no effect on CSFV RNA replication and extracellular titers of progeny virus (Figure 8). These results demonstrated that IL-8 production did not contribute to CSFV replication in SUVECs, suggesting that NS4A may promote CSFV replication in an IL-8-independent manner. Thus, CSFV NS4A may enhance CSFV replication through other means, such as whether NS4A regulates NS5A phosphorylation or promotes the viral replication complex formation to promote CSFV replication. This will be further verified in future studies. However, we demonstrated that NS4A promoted CSFV replication.

In conclusion, this study demonstrates for the first time that CSFV infection induces IL-8 production through MAVS pathway and production of ROS, and CSFV NS4A induces IL-8 production through enhancing MAVS pathway and promotes CSFV replication. This work lays the foundation to further understand the molecular mechanisms of IL-8 regulation and NS4A functions during CSFV infection.

## Authors contributors

WD and HL: collected data, performed all the experiments and wrote this manuscript; KG: revised the manuscript; TW, YO, and MJ: helped to finish the experiments; YZ: designed the experiments; All authors discussed the results and approved the final version.

### Conflict of interest statement

The authors declare that the research was conducted in the absence of any commercial or financial relationships that could be construed as a potential conflict of interest.
